# N-Acetylcysteine and Acetylsalicylic Acid Inhibit Alcohol Consumption by Different Mechanisms: Combined Protection

**DOI:** 10.3389/fnbeh.2020.00122

**Published:** 2020-07-31

**Authors:** María Elena Quintanilla, Fernando Ezquer, Paola Morales, Marcelo Ezquer, Belen Olivares, Daniela Santapau, Mario Herrera-Marschitz, Yedy Israel

**Affiliations:** ^1^Molecular and Clinical Pharmacology Program, Institute of Biomedical Sciences, Faculty of Medicine, University of Chile, Santiago, Chile; ^2^Centro de Medicina Regenerativa, Facultad de Medicina Clínica Alemana, Universidad del Desarrollo, Santiago, Chile; ^3^Department of Neuroscience, Faculty of Medicine, University of Chile, Santiago, Chile; ^4^Centro de Química Médica, Facultad de Medicina Clínica Alemana, Universidad del Desarrollo, Santiago, Chile

**Keywords:** N-acetylcysteine, aspirin, ethanol, relapse, rats

## Abstract

Chronic ethanol intake results in brain oxidative stress and neuroinflammation, which have been postulated to perpetuate alcohol intake and to induce alcohol relapse. The present study assessed the mechanisms involved in the inhibition of: (i) oxidative stress; (ii) neuroinflammation; and (iii) ethanol intake that follow the administration of the antioxidant N-acetylcysteine (NAC) and the anti-inflammatory acetylsalicylic acid (ASA) to animals that had consumed ethanol chronically. At doses used clinically, NAC [40 mg/kg per day orally (p.o.)] and ASA (15 mg/kg per day p.o.) significantly inhibited chronic alcohol intake and relapse intake in alcohol-preferring rats. The coadministration of both drugs reduced ethanol intake by 65% to 70%. N-acetylcysteine administration: (a) induced the Nrf2-ARE system, lowering the hippocampal oxidative stress assessed as the ratio of oxidized glutathione (GSSG)/reduced glutathione (GSH); (b) reduced the neuroinflammation assessed by astrocyte and microglial activation by immunofluorescence; and (c) inhibited chronic and relapse ethanol intake. These effects were blocked by sulfasalazine, an inhibitor of the xCT transporter, which incorporates cystine (precursor of GSH) and extrudes extracellular glutamate, an agonist of the inhibitory mGlu2/3 receptor, which lowers the synaptic glutamatergic tone. The inhibitor of mGlu2/3 receptor (LY341495) blocked the NAC-induced inhibition of both relapse ethanol intake and neuroinflammation without affecting the GSSG/GSH ratio. Unlike N-acetylcysteine, ASA inhibited chronic alcohol intake and relapse *via* lipoxin A4, a strong anti-inflammatory metabolite of arachidonic acid generated following the ASA acetylation of cyclooxygenases. Accordingly, the lipoxin A4 receptor inhibitor, WRW4, blocked the ASA-induced reduction of ethanol intake. Overall, *via* different mechanisms, NAC and ASA administered in clinically relevant doses combine their effects inhibiting ethanol intake.

## Introduction

The proposal put forward by Quertemont et al. (2005) that acetaldehyde generated in the metabolism of ethanol by brain catalase would play a role in eliciting the reinforcing effects of ethanol has been supported by a number of studies. Reports from Canada, Spain, United States, Italy, and Chile have shown that: (i) the administration of inhibitors of catalase or acetaldehyde trapping agents inhibits alcohol self-administration in animals (Aragon and Amit, 1992; Koechling and Amit, 1994; Tampier et al., 1995; Orrico et al., 2017; Peana et al., 2015); (ii) rats self-administer acetaldehyde into the brain ventral tegmental area (VTA), at concentrations that are three orders of magnitude lower than those required for ethanol to induce its self-administration (Rodd et al., 2005); and (iii) a VTA-directed gene transduction aimed at either inhibiting catalase synthesis or overexpressing the low K_m_ aldehyde dehydrogenase 2 (ALDH2) virtually blocks the self-administration of ethanol (Karahanian et al., 2011, 2015). Additionally, (iv) it has been shown that salsolinol, the adduct formed between acetaldehyde and dopamine, likely mediates the early reinforcing effects of acetaldehyde (Quintanilla et al., 2014; Melis et al., 2015; Quintanilla et al., 2016a; Rodd et al., 2003; Rojkovicova et al., 2008). The reader is referred to a comprehensive review on the actions of acetaldehyde (Correa et al., 2012).

The logical extension that inhibiting the molecular mechanisms that initiate ethanol intake would also inhibit chronic alcohol intake proved to be wrong; several studies showed that ethanol intake by rats that had learned to chronically self-administer ethanol was not inhibited by either: (a) anticatalase inhibitors (Peana et al., 2015); (b) acetaldehyde trapping agents (Peana et al., 2015; Orrico et al., 2017); or (c) anticatalase gene transduction or Aldh2 gene overexpression (Quintanilla et al., 2012; Karahanian et al., 2015).

From the above reports, mechanism(s) different from those that initiate the self-administration of ethanol must account for this “autopilot” continuation of alcohol intake. For several addictive drugs, relapse self-administration has been reported to be inhibited by the antioxidant drug N-acetylcysteine (NAC; Duailibi et al., 2017; Garcia-Keller et al., 2019). For alcohol, studies by Quintanilla et al. (2016a, 2018), Lebourgeois et al. (2018, 2019), and Israel et al. (2019) showed that the administration of NAC markedly inhibits both chronic alcohol intake and relapse intake. Treatments with NAC were shown to significantly inhibit both oxidative stress and neuroinflammation (Quintanilla et al., 2018; Israel et al., 2019).

The above studies dovetail with studies from the laboratories of Crews, Guerri, and Harris, who showed that chronic alcohol intake or its administration leads to neuroinflammation (Alfonso-Loeches et al., 2010; Mayfield et al., 2013; Crews and Vetreno, 2016). Neuroinflammation and oxidative stress highly interrelate and self-perpetuate each other (Schreck et al., 1992; Schulze-Osthoff et al., 1992; Canty et al., 1999; Kastl et al., 2014; Israel et al., 2019). Studies by Qin et al. (2007) showed that the administration of a single systemic dose of lipopolysaccharide (LPS) results in brain inflammation that remains for many months. Studies by Blednov et al. (2011) showed that the administration of LPS to mice increased the preference for increasing concentrations of alcohol solutions, an effect that was long-lasting. Noteworthy, oral alcohol intake (yielding acetaldehyde generated in the gut) leads to an increased gut leakiness, which allows bacterial LPS to enter into the portal circulation (Ferrier et al., 2006). Noteworthy, in detoxified alcoholics, a strong relationship is seen between plasma LPS and proinflammatory cytokine levels and alcohol craving (Leclercq et al., 2017).

While NAC has been used for decades as a strong antioxidant, the actual mechanism of its antioxidant effect is not fully clear. Recent studies showed that NAC amide (NACA), an analog of NAC, attenuated the oxidative stress in rats following traumatic brain injury *via* activation of the nuclear factor erythroid 2-related factor 2 (Nrf2)–antioxidant response element (ARE) signal pathway (Zhou et al., 2018). *In vitro*, an overexpression of the Nrf2 system has been shown to up-regulate the activity of the promoter of the cystine/glutamate antiporter xCT in several types of cells (Habib et al., 2015; Shih et al., 2003). Thus, NAC *via* Nrf2 could reduce the glutamatergic tone both by activation of the xCT cystine-glutamate antiporter and increasing cystine as substrate for the antiporter, thus activating the inhibitory presynaptic metabotropic mGlu2/3 receptor.

Recent studies in alcohol-preferring rats showed that both neuroinflammation and chronic alcohol intake/relapse are inhibited by administration of acetylsalicylic acid (ASA; Israel et al., 2019). These effects of ASA were accompanied by increases in the levels of the glutamate transporter GLT-1 (see also Romera et al., 2007; Sobrado et al., 2009). Increases in GLT-1 levels and a reduction of chronic ethanol intake following the administration of β-lactam antibiotics have also been reported by Sari and associates in rats selected as alcohol drinkers (Rao et al., 2015; Sari et al., 2016). Recent and previous studies also indicate that the β-lactam ceftriaxone displays anti-inflammatory effects (Amin et al., 2012; Ochoa-Aguilar et al., 2018).

A number of studies have shown that ASA, even at low anticlotting doses, acutely acetylates cyclooxygenase-1 (Cox-1) in platelets, and chronically, it also acetylates Cox-2 leading to the generation of a powerful anti-inflammatory agent; namely, 15-R epi-lipoxin A4 (lipoxin A4 or ATL), a metabolite of arachidonic acid (Romano et al., 2015; Serhan and Levy, 2018). Whether the inhibitory effect of ASA on ethanol intake is due to the generation of lipoxin A4 is not known.

In operant self-administration studies, the presentation of a stimulus previously associated with alcohol self-administration leads to marked increases in glutamate release in nucleus accumbens (Gass et al., 2011). An increased release of glutamate is postulated to play an important role in addictive drug relapse and craving (see Scofield et al., 2016). Thus, it is hypothesized that increases in the GLT-1 transporter, as reported for ASA (Israel et al., 2019), added to an increase in an xCT-mediated presynaptic mGlu2/3 inhibitory tone induced by NAC, should provide greater inhibitory effect on alcohol intake when these drugs are administered together.

In the present studies, we investigated whether a reduction of ethanol intake induced by NAC follows the activation of the hippocampal Nrf2-ARE antioxidant signaling pathway, which could be prevented by inhibition of the xCT cystine-glutamate transporter and by inhibition of the glutamate presynaptic metabotropic mGlu2/3 receptor. Further, we evaluated whether blocking the lipoxin A4 receptor blunts the inhibition of ethanol intake and alcohol relapse intake exerted by ASA.

## Materials and Methods

### Animals

Adult female Wistar-derived rats, selectively bred for over 90 generations as alcohol consumers (University of Chile Bibulous; UChB; Quintanilla et al., 2006; Israel et al., 2017), were used in the experiments. Animals were maintained on a 12-h light–dark cycle (lights off at 7:00 PM) and regularly fed a soy protein, peanut-meal rodent diet (Cisternas, Santiago, Chile). Female rats were used because females maintain stable body weights (within 10%) over time, which is of value in long-term studies. Furthermore, Guerri and associates (Pascual et al., 2017) showed that female mice were more susceptible than males to develop an ethanol-induced neuroinflammation. Experimental procedures were approved by the Ethics Committee for Experiments with Laboratory Animals at the Medical Faculty of the University of Chile (protocol CBA# 0994 FMUCH) and by the Chilean Council for Science and Technology Research.

### Drugs

Alcohol solutions were prepared from absolute graded ethanol (Merck, Darmstadt, Germany) diluted to 10% or 20% alcohol solutions (vol/vol) in tap water. N-acetylcysteine (Sigma–Aldrich, St. Louis, MO, USA) was dissolved in saline, adjusted with NaOH to pH 7.2, and administered in volume of 5.0 ml/kg per day. Acetylsalicylic acid (Sigma–Aldrich, St. Louis, MO, USA) was dissolved in water and adjusted with NaOH to pH 7.2. To study the effect of the combination of NAC + ASA on chronic ethanol intake (Experiment 1), NAC was administered i.p. for 2 days at a loading dose of 70 mg/kg per day (Israel et al., 2019), each dose being one-half of the loading dose (140 mg/kg) administered clinically in the treatment of acetaminophen hepatotoxicity (Fisher and Curry, 2019), followed by a lower maintenance dose of 40 mg/kg per day (by oral gavage) administered for 11 days. The maintenance dose of NAC chosen (40 mg/kg per day) was similar to that used in humans for cocaine treatment studies (Nocito Echevarria et al., 2017). Acetylsalicylic acid was administered at a dose of 15 mg/kg per day, by oral gavage (Israel et al., 2019), for 13 days. This dose is considerably lower than that used as chronic treatment for arthritis (Colebatch et al., 2012). Doses of ASA of 15–30 mg/kg have been shown not to generate gastric irritation in rats (Wallace et al., 2004). When the combination of NAC + ASA was administered, both drugs were dissolved in water, adjusted with NaOH to pH 7.2, and given in a volume of 5 ml/kg per day by oral gavage, for 11 days. In the three subsequent experiments, aimed at identifying the underlying mechanisms involved in the effect of NAC and ASA on alcohol relapse (Figures 3, 7, 8), we used a shorter treatment period (2 or 4 days) and a higher concentration of NAC (100 mg/kg per daily) and ASA (15 mg/kg per day, Figure 7; and 30 mg/kg per day, Figure 8) both administered p.o. Other drugs were administered parenterally to achieve a better bioavailability at the time of administration of NAC or ASA. Their schedules of administration are described for each experiment. The mGlu2/3 receptor inhibitor LY341495 disodium salt (Tocris Cookson Limited, Bristol, UK) was dissolved in 66 mM phosphate-saline buffer (pH 8.0) and administered intraperitoneally (i.p.) at a dose of 1 mg/kg per day (delivered as 5.0 ml/kg), as previously reported (Fukumoto et al., 2014). The xCT transporter inhibitor sulfasalazine (SZ; Sigma-Aldrich; cat. no. SO883) stock solution was prepared dissolving sulfasalazine (SZ) in dimethyl sulfoxide (80 mg/ml), as previously reported (Bernabucci et al., 2012). Fresh sulfasalazine was prepared every day by dilution of the stock solution in isotonic saline and administered i.p. at a dose of 8 mg/kg per day delivered as 5 ml/kg.

**Figure 1:**
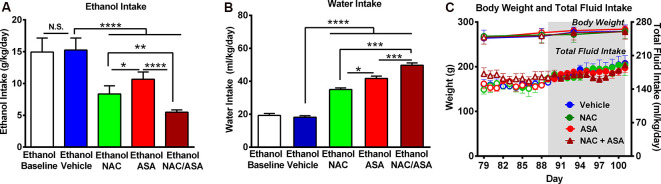
Administration of N-acetylcysteine (NAC; 40 mg/kg per day p.o.) + acetylsalicylic acid (ASA; 15 mg/kg per day p.o.) marked reduced ethanol intake in rats that had chronically consumed ethanol. **(A)** Significant inhibition of chronic alcohol intake following the oral administration of NAC or ASA vs. vehicle group [one-way analysis of variance (ANOVA): *F*_4, 25_ = 46.53, ****P < 0.0001; Tukey *post hoc*: vehicle-treated vs. NAC-treated and vs. ASA-treated groups, ****P < 0.0001, *n* = 6 rats per group]. Inhibition of ethanol intake was further increased by the coadministration of NAC + ASA when compared with either NAC or ASA group (**P < 0.01, NAC + ASA–treated vs. NAC- treated group; ****P < 0.0001, NAC + ASA–treated vs. ASA-treated group). Bars represent mean ± SEM of 11 consecutive days of ethanol intake, during which rats received by oral gavage NAC, ASA, NAC + ASA, or vehicle (from day 91 to day 101 of ethanol intake). Chronic ethanol intake of all groups (10-day baseline) prior to vehicle administration (mean ± SEM, 15.0 ± 0.9 g ethanol/kg per day) was not different from that of rats that received vehicle (mean ± SEM, 15.3 ± 0.8 g ethanol/kg per day; A: white bar vs. blue bar). Additionally, we found no significant differences between the baseline ethanol intakes of the four groups prior to drug/vehicle treatment [mean ± SEM, 15.0 ± 0.80 (ethanol vehicle); 14.0 ± 1.4 (ethanol NAC); 14.0 ± 0.2 (ethanol ASA); and 14.0 ± 0.7 (ethanol NAC + ASA) g ethanol/kg per day, *n* = 6/group; ANOVA: *F*_3, 20_ = 0.2968, *P*: N.S.] **(B)** NAC, ASA, NAC + ASA markedly increased water intake compared with the control (ethanol vehicle) and baseline group (ANOVA: *F*_4, 25_ = 132.5, *P* < 0.0001). **(C)** The administration of NAC, ASA, or the combination of NAC and ASA did not affect total fluid intake (ANOVA: *F*_3, 48_ = 1.406, *P*: N.S.) or body weight- (ANOVA: *F*_3, 12_ = 0.416, P: N.S) compared with vehicle administration. *P < 0.05 means significant difference between Ethanol NAC and Ethanol ASA groups. ***P < 0.001 means significant differences between Ethanol NAC/ASA with Ethanol NAC and Ethanol ASA groups. N.S., not statistically significant.

**Figure 2:**
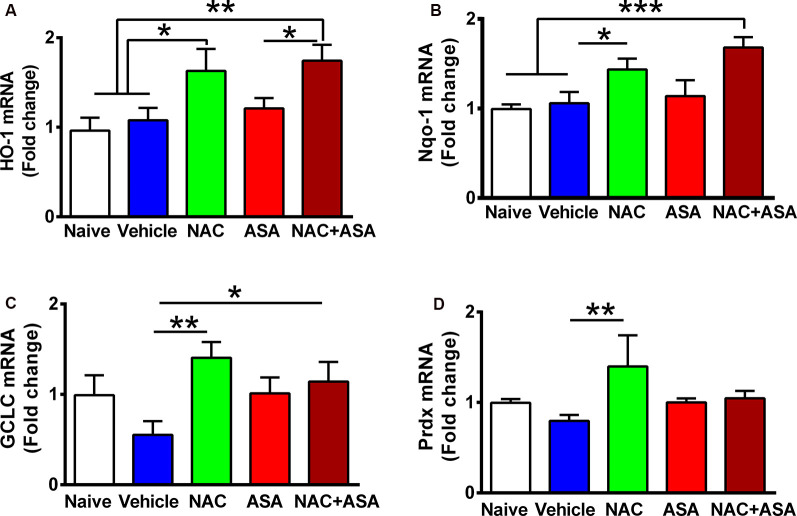
Oral administration of N-acetylcysteine (NAC; 40 mg/kg per day), but not acetylsalicylic acid (ASA; 15 mg/kg per day), increased the expression of Nrf2-ARE regulated genes in the hippocampus of chronically alcohol-consuming rats. Reduction of chronic ethanol intake induced by the oral administration of NAC (40 mg/kg per day) shown in Figure 1 was associated with a significant increase in the hippocampal level of Nrf2-ARE regulated genes compared to chronic ethanol drinking rats receiving vehicle or compared to naive rats that had ingested only water. A one-way analysis of variance (ANOVA) performed on ethanol intake data revealed significant effect of treatment on **(A)** heme oxygenase 1 (HO-1) mRNA levels (ANOVA *F*_(4, 24)_ = 6.417, **P < 0.01); Tukey *post hoc* test revealed that rats treated with NAC or NAC + ASA showed in the hippocampus a greater mRNA expression of HO-1 vs. vehicle group (*P < 0.05 vehicle-treated vs. NAC- and **P < 0.01 vs. NAC + ASA-treated groups). **(B)** Quinone oxidoreductase 1 (Nqo1) levels. ANOVA *F*_treatment(4, 26)_ = 5.332, **P < 0.01. *Post hoc* test revealed that rats treated with NAC or NAC + ASA showed greater mRNA expression of Nqo1 vs. vehicle group (*P < 0.05 vehicle-treated vs. NAC-treated group, ***P < 0.001 vehicle-treated and naive vs. NAC + ASA-treated group). **(C)** γ-Glutamyl-cysteine synthetase (GCLC) mRNA levels. ANOVA *F*_treatment(4, 26)_ = 2.852, **P < 0.01. *Post hoc* test revealed rats treated with NAC or NAC + ASA showed greater mRNA expression of GCLC vs. vehicle group (**P < 0.01 vehicle-treated vs. NAC-treated group, *P < 0.05 vehicle-treated vs. NAC + ASA–treated group) and **(D)** peroxiredoxin (Prdx) mRNA levels. ANOVA *F*_treatment(4, 26)_ = 2.912. *Post hoc* test revealed rats treated with NAC showed greater mRNA expression of Prdx vs. vehicle group (**P < 0.01 vehicle-treated vs. NAC-treated group). Data of each target gene were normalized by the expression of the housekeeping gene glyceraldehyde-3-phosphate dehydrogenase (GAPDH) in the same sample. Data are presented as mean ± SEM, *n* = 6 rats per experimental group.

**Figure 3:**
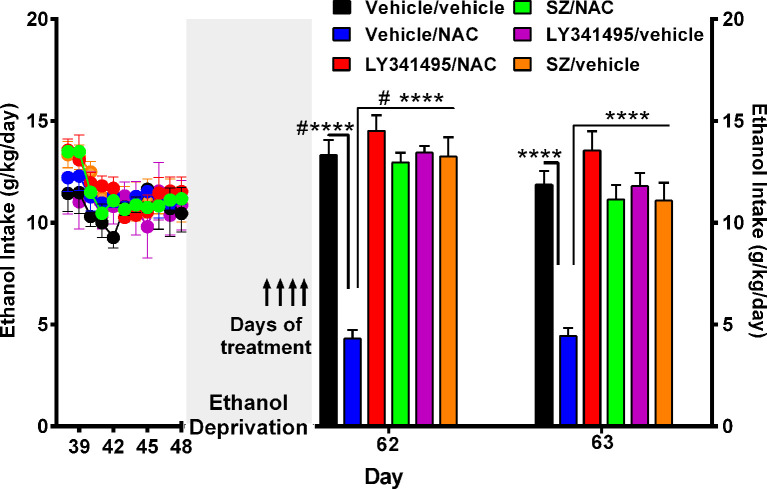
N-acetylcysteine (NAC)–induced inhibition of alcohol relapse-like drinking is prevented by blockade of the glutamate mGlu2/3 receptors (LY3441495) and the xCT-cystine-glutamate exchanger (sulfasalazine, SZ). Following 48 days of chronic ethanol intake and a subsequent ethanol deprivation for 2 weeks, rats received their respective treatment for only the last 4 days of deprivation (arrows in Figure). Given the short administration period, the dose of NAC was increased to 100 mg/kg per day. Ethanol reaccess was allowed 24 h after the last dose of SZ or LY3441495. Following the 2 weeks of alcohol deprivation and ethanol reaccess, the high ethanol intake of the vehicle/vehicle group (black bars) on days 62 and 63 demonstrate the alcohol deprivation relapse. Two-way analysis of variance (treatment × day) performed on ethanol intake data obtained the first (day 62) and second (day 63) days of ethanol reaccess post deprivation revealed significant effect of treatment (*F*_treatment(5,46)_ = 31.0, ****P < 0.0001), day (*F*_day(1,46)_ = 21.73, ****P < 0.0001), and significant treatment × day interaction (*F*_interaction(5, 46)_ = 20.65, ****P < 0.0001). Tukey *post hoc* analysis indicated that NAC (vehicle/NAC) induced a significant reduction of alcohol relapse drinking (****P < 0.0001 vehicle/NAC group compared to vehicle/vehicle group), whereas pretreatment with LY341495, an antagonist of the mGlu2/3 receptors, or sulfasalazine (SZ), an inhibitor of the xCT-cystine/glutamate exchanger, fully blocked the NAC-induced reduction of alcohol relapse (****P < 0.0001 LY341495/NAC group vs. vehicle/NAC group; and ****P < 0.0001 SZ/NAC group vs. vehicle/NAC group). However, sulfasalazine at the dose of 8 mg/kg per day administered without NAC (SZ/vehicle group) did not *per se* inhibit ethanol intake the first or second reaccess day; nor did it show a significant anti-inflammatory effect (*vide infra* Figure 6) vs. the vehicle/vehicle group (Tukey *post hoc* SZ/vehicle vs. vehicle/vehicle group: N.S.). Similarly, LY341495 at a dose of 1 mg/kg per day administered without NAC (LY341495/vehicle group) did not *per se* inhibit ethanol intake the first or second reaccess day; nor did it show a significant anti-inflammatory effect (*vide infra* Figure 6) vs. the vehicle/vehicle group (Tukey *post hoc* LY341495/vehicle vs. vehicle/vehicle group: N.S.). In addition, sulfsalazine or LY341495 treatments did not influence total fluid intake. Furthermore, 24 h after the administration of sulfasalazine or LY341495, rats were given reaccess to ethanol to recorder their consumption. Data are presented as mean ± SEM, *n* = 6 rats per experimental group. ^#^*P* < 0.05 means significant difference with the same group before deprivation. N.S., not statistically significant.

**Figure 4:**
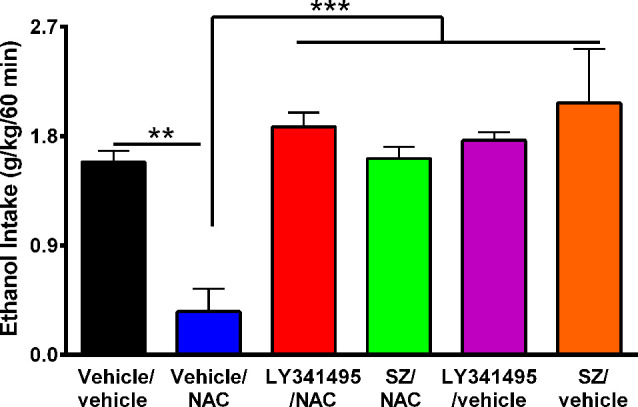
The N-acetylcysteine (NAC)–induced inhibition of binge-like ethanol intake in the first 60 min of ethanol reaccess was fully blocked by inhibition of the glutamate mGlu2/3 receptor or the xCT-cystine/glutamate exchanger. Data showed ethanol intake of all groups during the first 60 min of ethanol reaccess. Analysis of variance of data indicates significant effect of treatments vs. vehicle/vehicle group (*F*_(5,23)_ = 11.56, ***P < 0.001). *Post hoc* analysis indicated that NAC (100 mg/kg per day for 4 days) induced a significant reduction of the alcohol relapse-like drinking (vehicle/NAC group compared to vehicle/vehicle group **P < 0.01), whereas pretreatment with LY341495, an antagonist of mGlu2/3 receptors, or sulfasalazine (SZ), an inhibitor of xCT-cystine/glutamate exchanger fully prevented the NAC-induced reduction of alcohol relapse (LY341495/NAC group vs. vehicle/NAC group ***P < 0.001 and SZ/NAC group vs. vehicle/NAC group ***P < 0.001). Data are presented as mean ± SEM, *n* = 6 rats per experimental group.

**Figure 5:**
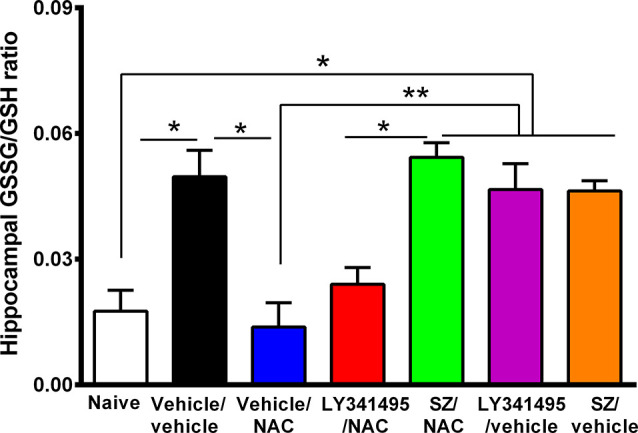
Marked increase in oxidative stress in the hippocampus of animals that had consumed ethanol chronically and were deprived for 14 days followed by 2 days of ethanol reaccess was reversed by the administration of N-acetylcysteine (NAC; 100 mg/kg for 4 days), an effect that was prevented by an inhibitor of xCT-cystine/glutamate exchanger. Oxidative stress determined as the GSSG/GSH ratio is markedly increased by chronic and post deprivation ethanol intake shown in Figure 4 [one-way analysis of variance (ANOVA): *F*_(6,28)_ = 10, *P* < 0.0001; Tukey *post hoc*: naive (water drinking) compared to ethanol vehicle/vehicle group, *P < 0.05]. In addition, NAC fully normalized the ethanol-induced increase in GSSG/GSH ratio (*post hoc*: vehicle/NAC-treated compared to vehicle/vehicle group *P < 0.05). Sulfasalazine (SZ), an inhibitor of xCT-cystine/glutamate exchanger, prevented the NAC-induced normalization of GSSG/GSH ratio (*post hoc*: vehicle/NAC-treated compared to SZ/NAC group **P < 0.01) but not an antagonist of mGlu2/3 receptor (LY341495/NAC vs. vehicle/vehicle group *P* > 0.05 N.S.). Data are presented as mean ± SEM, *n* = 6 rats per experimental group. N.S., not statistically significant.

**Figure 6:**
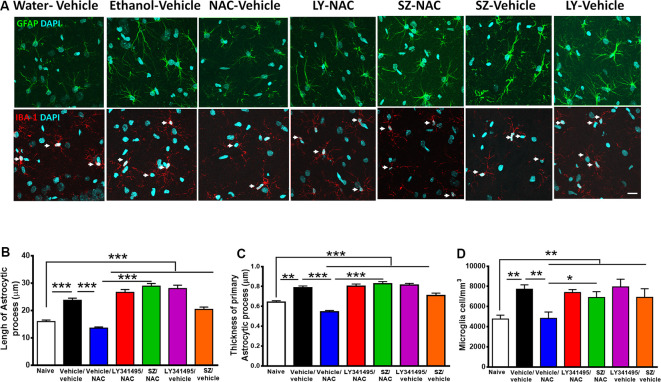
Alcohol-induced neuroinflammation is inhibited by N-acetylcysteine (NAC; 100 mg/kg per day): effect blocked by inhibitors of xCT-transporter and mGlu2/3 receptor. (**A**, Top) Astrocyte immunofluorescence (GFAP immunoreactivity, green; DAPI, blue; A, bottom: microglia immunofluorescence (IBA-1 immunoreactivity, red; DAPI, blue, depicted by white arrows). Scale Bar 25 μm. Rats ingested alcohol chronically for 48 days followed by 14 days of ethanol deprivation and further 2 days of ethanol reaccess. Alcohol-treated rats treated with vehicle (vehicle/vehicle group) led to an increase in length (analysis of variance: *F*_6, 1531_ = 55.61, ^***^P < 0.001; Tukey *post hoc*: naive compared to vehicle/vehicle-treated group ^***^P < 0.001) and thickness (*F*_6, 733_ = 35.77, ^***^P < 0.001; Tukey *post hoc*: naive compared to vehicle/vehicle-treated group **P < 0.01) of astrocyte processes **(B,C)** and to an increase in microglial density (*F*_6, 44_ = 4.543; **P < 0.01; Tukey *post hoc*: naive compared to vehicle/vehicle-treated group *P < 0.05; **D**) compared to (naive) rats drinking water. The administration of NAC (vehicle/NAC) fully normalized the ethanol-induced increase in astrocyte length (Tukey *post hoc*: vehicle/vehicle-treated compared to vehicle/NAC-treated group ^***^P < 0.001) and thickness (*post hoc*: vehicle/vehicle-treated compared to vehicle/NAC-treated group ^***^P < 0.001). N-acetylcysteine also blocked the ethanol-induced increase in microglial density (**D**; Tukey *post hoc*: vehicle/vehicle-treated compared to vehicle/NAC-treated group *P < 0.05). Pretreatment prior to ethanol reaccess with LY341495, an antagonist of glutamate mGlu2/3 receptors and SZ, an inhibitor of xCT-cystine/glutamate exchanger, prevented the NAC-induced normalization of length (vehicle/NAC-treated compared to LY341495Y/NAC-treated group ^***^P < 0.001 and vs. SZ/NAC-treated group ^***^P < 0.001) and thickness (vehicle/NAC-treated compared to LY341495Y/NAC-treated group ^***^P < 0.001; and compared to SZ/NAC-treated group ^***^P < 0.001) of astrocytic process. Pretreatment with LY341495 or sulfasalazine also blocked the effect of NAC on microglial density (vehicle/NAC-treated compared to LY341495Y/NAC-treated treated group *P < 0.05, and compared to SZ/NAC-treated group *P < 0.05). Data are presented as mean ± SEM, *n* = 6 rats per experimental group.

**Figure 7:**
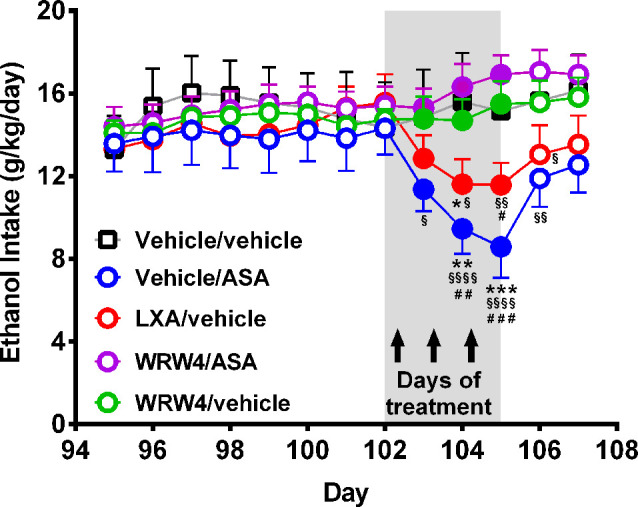
Oral administration of acetylsalicylic acid (ASA) or intravenous administration of lipoxin A4 inhibited chronic ethanol intake an effect that was fully blocked by the administration of WRW4, an antagonist of the lipoxin FPR2/ALX receptor. Rats chronically ingesting ethanol for 102 days were treated for three consecutive days with either: vehicle, ASA (15 mg/kg, p.o.), lipoxin A4 (LXA; 7 μg/kg, i.v.), or WRW4 (0.3 mg/kg administered i.v. 5 min prior to ASA or vehicle). Acetylsalicylic acid or LXA A4 treatment reduced chronic ethanol intake compared to the vehicle group [two-way analysis of variance indicates significant effect of treatment (*F*_treatment(4, 108)_ = 14.23, ^***^P < 0.001) but not of day (*F*_day(5, 108)_ = 1.262, *P* = 0.2856)]. Tukey *post hoc* test revealed that the administration of three daily doses of ASA reduced ethanol intake: vs. the vehicle group (**P < 0.01, the second and ^***^P < 0.001 the third day of treatment), vs. the WRW4/ASA group (^§^*P* < 0.05 the first, ^§§§^*P* < 0.001 the second, ^§§§§^*P* < 0.0001 the third day of treatment, and ^§§^*P* < 0.01 the first posttreatment day) and vs. the WRW4/vehicle group (^##^*P* < 0.01, the second and ^###^*P* < 0.01 the third day of treatment). In the same way, three daily doses of lipoxin A4 reduced ethanol intake: vs. vehicle group (*P < 0.05, the second day of treatment), vs. the WRW4/ASA group (^§^*P* < 0.05 the second, ^§§^*P* < 0.01 the third day of treatment, and ^§§^*P* < 0.01 the first posttreatment day) and vs. the WRW4/vehicle group (^#^*P* < 0.05, the third day of treatment). Data are presented as mean ± SEM, *n* = 6 rats per experimental group.

**Figure 8:**
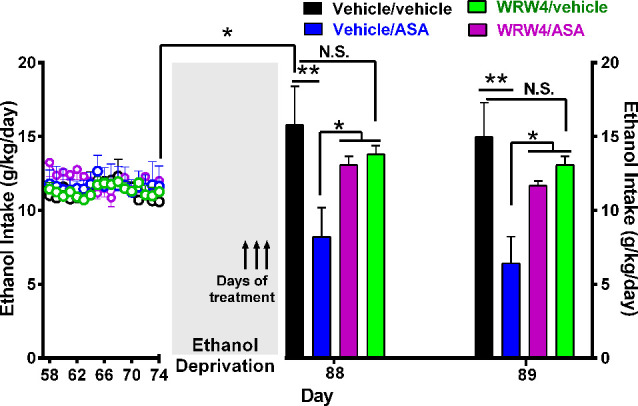
Oral administration of acetylsalicylic acid (ASA, 30 mg/kg per day) inhibited the alcohol postdeprivation relapse drinking, an effect blocked by the administration of WRW4, an antagonist of the lipoxin FPR2/ALX receptor. Following 74 days of ethanol intake, on the last 3 days of a 14-day ethanol deprivation, animals were treated as indicated by arrows. Bars show daily ethanol intake after the 2-week ethanol deprivation followed by 2 days of ethanol reaccess. The high ethanol intake of the control group (vehicle/vehicle group; black bar) on the first day (day 88) of alcohol reaccess (16.0 ± 2.0; mean ± SEM, *n* = 5) vs. its intake prior to deprivation (11.2 ± 0.34; mean ± SEM, *n* = 5) demonstrates the alcohol deprivation relapse [*P < 0.05 vehicle/vehicle group on the first day of ethanol re-exposure (black bar) compared to the mean of the last 11 baseline days of ethanol intake of the same group (empty black circles)]. Two-way analysis of variance of (treatment × day) performed on ethanol intake data obtained the first (day 88) and second (day 89) day of ethanol reaccess postdeprivation revealed significant effect of treatment (*F*_treatment(3, 26)_ = 9.315), but not of day.* Post hoc* Fisher analysis indicated that ASA (vehicle/ASA group) administration 24 h before ethanol reaccess induced a significant (**P < 0.01) inhibition of ethanol intake vs. the vehicle/vehicle group the first and second day of ethanol reaccess, whereas pretreatment with WRW4, an antagonist of the lipoxin receptor (FPR2), blocked the ASA-induced reduction of alcohol relapse the first and second day of ethanol reaccess (*P < 0.05 vehicle/ASA group vs. WRW4/ASA and WRW4/vehicle group). Data are presented as mean ± SEM, *n* = 6 rats per experimental group. N.S., not statistically significant.

Lipoxin A4 (stock ethanol solution 25 μg/250 μl; Cayman Chemical, Ann Arbor, MI, USA), an ASA-triggered endogenous lipid mediator with potent anti-inflammatory properties (Medeiros et al., 2013), which acts as an agonist of the formyl peptide receptor 2 (FPR2/ALX), a lipoxin receptor, was prepared by dilution of the stock ethanol solution in isotonic saline and administered at a dose of 7 μg/kg delivered as 1 ml/kg intravenously (i.v.), as recommended (Wu et al., 2013). WRW4 (Tocris Bioscience Reagent, Bristol, UK; cat. no. 2262), an antagonist of the FPR2/ALX receptor, was dissolved in isotonic saline and administered at a dose of 0.3 mg/kg per day, delivered as 1 ml/kg, into the tail vein.

### Brain Tissue Samples

We selected the whole left hippocampus for oxidized glutathione (GSSG)/reduced glutathione (GSH) and reverse transcriptase–quantitative polymerase chain reaction (RT-qPCR) studies. Right hippocampus was used for immunohistochemical studies to evaluate astrocyte and microglial reactivity focusing on the stratum radiatum of CA1 region according to our previous studies (Ezquer et al., 2019; Israel et al., 2019) and according to Gómez et al. (2018).

### Quantification of mRNA Levels of Nrf2 Regulated Genes in Hippocampus

Twenty-four hours after the oral administration of NAC, ASA, NAC + ASA, or saline, for 11 consecutive days, chronically ethanol-consuming rats were anesthetized with chloral hydrate (280 mg/kg, i.p.) and euthanized to obtain hippocampal samples, as indicated above. Total RNA was isolated using Trizol (Invitrogen, Grand Island, NY, USA). One microgram of total RNA was used to perform reverse transcription with MmlV RT (Invitrogen) and oligo dT primers. Real-time PCR reactions were performed to amplify the Nrf2 activated genes heme oxygenase 1 (HO-1), NAD(P)H:quinone oxidoreductase 1 (Nqo1), peroxiredoxin (Prdx), and γ-glutamyl-cysteine synthetase (GCLC) using a Light-Cycler 1.5 thermocycler (Roche, Indianapolis, IN, USA). To ensure that amplicons were generated from mRNA and not from genomic DNA, controls without RT during the reverse transcription reaction were included. Relative quantification was performed using the ΔΔCT method. The mRNA level for each target gene was normalized against the mRNA level shown by the housekeeping gene glyceraldehyde-3-phosphate dehydrogenase (GAPDH) in the same sample. The primers used for qPCR amplifications were designed by the authors: HO-1 forward: 5′-CTATCGTGCTCGCATGAAC-3′; HO-1 reverse: 5′-CAGCTCCTCAAACAGCTCAA-3′; Nqo1 forward: 5′-CTCGCCTCATGCGTTTTTG-3′; Nqo1 reverse: 5′-CCCCTAATCTGACCTCGTTCAT-3′; GCLC forward: 5′-CTGAGGCAAGATACCTTTATGACC-3′; GCLC reverse: 5′-GTAGCTATCTATTGAGTCATACCGAGAC-3′; Prdx forward: 5′-GGAGGATTGGGACCCATGAAC-3′; Prdx reverse: 5′-AGAGCGGCCAACAGGAAGATC-3′; GAPDH forward: 5′-GACATGCCGCCTGGAGAAAC-3′; GAPDH reverse: 5′-AGCCCAGGATGCCCTTTAGT-3′.

### Glutathione Determination

Following continuous chronic ethanol intake for 48 days, animals were alcohol deprived for 14 days and treated on the last 4 days of deprivation with either LY341495/NAC, SZ/NAC, LY341495/vehicle, SZ/vehicle, or vehicle/vehicle and then offered ethanol access for two consecutive days to evaluate alcohol relapse intake. Water was also available. Immediately, after the second day of reaccess to ethanol (after the alcohol reading at day 63, the animals were anesthetized with chloral hydrate (280 mg/kg, i.p.) and euthanized to obtain hippocampal samples. Brain oxidative stress was determined as the GSSG/GSH ratio in hippocampus as previously described (Ezquer et al., 2019). Glutathione reductase (cat. no. G3664), NADPH (cat. no. N1630), and DTNB (5,50-dithiobis-2-nitrobenzoic acid; cat. no. D-8130) were used for the determination of glutathione, purchased from Sigma-Aldrich.

### Determination of Astrocyte and Microglia Immunoreactivity

Following continuous chronic ethanol intake for 48 days, animals were alcohol deprived for 14 days and treated for the last 4 days of deprivation with either LY341495/NAC, SZ/NAC, LY341495/vehicle, SZ/vehicle, or vehicle/vehicle and then offered ethanol access for two consecutive days. Water was always available. Immediately, after the second day of reaccess to ethanol, the animals were anesthetized with chloral hydrate (280 mg/kg, i.p.) and euthanized to obtain hippocampal samples. Immunofluorescence against the astrocyte marker glial fibrillary acidic protein (GFAP) and the microglial marker ionized-calcium–binding adaptor molecule 1 (Iba-1) was evaluated in coronal cryosections of hippocampus (30 μm thick) as previously reported (Ezquer et al., 2019). Nuclei were counterstained with DAPI. Microphotographs were taken from the stratum radiatum of hippocampus using a confocal microscope (Olympus FV10i). The area analyzed for each stack was 0.04 mm^2^, and the thickness (*z* axis) was measured for each case. The total length and thickness of GFAP-positive primary astrocytic processes and density of Iba-1–positive microglial cells were assessed using FIJI image analysis software (http://fiji.sc/Fiji) as previously reported (Ezquer et al., 2019).

### Experiment 1: Effect of Oral Administration of NAC, ASA, or Their Combination on Chronic Alcohol Intake and mRNA Expression of Nrf2-ARE Regulated Genes in the Hippocampus

Twenty-four female UChB rats, weighing 190–210 g were single housed in cages at the age of 60 days and maintained on a 12-h light-dark cycle (lights off at 7:00 PM). Subsequently, rats received continuous concurrent free-choice access to 10% (vol/vol) ethanol solution and water for 77 days. On day 78, rats were allowed to concurrent three-bottle choice access of 10% and 20% (vol/vol) ethanol solutions and water for 24 additional days. On day 88, rats were divided in four groups: (*n* = 6 per group), namely, (1) vehicle group: rats were given saline on the first 2 days by the i.p. route and water by oral gavage for the following 11 days; (2) NAC group: On the first 2 days, NAC was administered by the i.p. route (70 mg/kg per day), as a loading dose, and subsequently by oral gavage (40 mg/kg per day) for the following 11 days; (3) ASA group: rats were given saline on the first 2 days by the i.p. route and thereafter administered ASA (15 mg/kg per day) by oral gavage, for the following 11 days; and (4) NAC + ASA (NAC + ASA) group: On the first 2 days, NAC was administered by the i.p. route (70 mg/kg per day), and subsequently, rats were daily administered the combined NAC (40 mg/kg per day) plus ASA (15 mg/kg per day) dose by oral gavage, for 11 days. Ethanol intake was recorded daily and expressed as grams of ethanol consumed/kg body weight for the complete 11-day period. Preliminary studies for this group of animals have been reported (Israel et al., 2019). Twenty-four hours after the last NAC, ASA, or NAC + ASA administration and saline controls, the animals were anesthetized with chloral hydrate (280 mg/kg, i.p.) to obtain hippocampal samples for quantification of mRNA levels of Nrf2-ARE regulated genes.

### Experiment 2. Effect of Oral Administration of NAC on Relapse-Like Ethanol Drinking: Role of Glutamate mGlu2/3 Receptor and of the xCT-Cystine/Glutamate Exchanger

Thirty-six female UChB rats, weighing 190–210 g, were single housed in cages at the age of 60 days and maintained on a 12-h light–dark cycle (lights off at 7:00 PM). Subsequently, rats received continuous concurrent free-choice access in the home cage to 10% (vol/vol) ethanol solution and water for 36 day. On day 37, rats were allowed to concurrent three-bottle choice access to 10% and 20% (vol/vol) ethanol solutions and water for 11 additional days. On day 48, after induction of chronic ethanol intake, animals were deprived of ethanol for 14 days and thereafter allowed reaccess to ethanol solutions (10% and 20%) and water, for 2 days. Water was always available through all the experiment. While the chronic ethanol ingestion period of the present experiment was shorter (47 days) than that of experiment 1, the rats showed a marked relapse intake to ethanol after two weeks of deprivation (alcohol reaccess on day 62), likely due to neuroinflammation and oxidative stress that self-perpetuate each other after ethanol is discontinued (see Israel et al., 2019). It is further noted that NAC does not inhibit alcohol intake if rats are allowed to drink ethanol for a short time (up to 18 days), whereas an inhibitory effect was clearly observed after 60 days of chronic intake (Quintanilla et al., 2016b).

On the last 4 days of the 14-day ethanol deprivation period, rats were divided into five groups (*n* = 6 rats/group): (1) LY341495/NAC group: rats were given LY341495, an inhibitor of the mGlu2/3 receptor, at a dose of 1 mg/kg per day (i.p.), 15 min before NAC administration (100 mg/kg per day by oral gavage (Fukumoto et al., 2014) for 4 days; (2) vehicle/NAC group: rats were given vehicle (isotonic saline) in a single dose (5 ml/kg; i.p.), 15 min before of NAC administration (100 mg/kg per day, by oral gavage); (3) SZ/NAC group: rats were given SZ, an inhibitor of xCT-cystine/glutamate exchanger, at a dose of 8 mg/kg per day (i.p.) 15 min before NAC administration (100 mg/kg per day, by oral gavage; Bernabucci et al., 2012); (4) LY341495/vehicle group: rats were given LY341495 at a dose of 1 mg/kg per day (i.p.), 15 min before vehicle administration (water 5 ml/kg per day, by oral gavage); (5) SZ/vehicle group: rats were given SZ (8 mg/kg per day i.p.) 15 min before vehicle administration (water 5 ml/kg per day, by oral gavage); or (6) vehicle/vehicle group: rats were given a single dose of isotonic saline *via* i.p. 15 min before a dose of vehicle (water by oral gavage). Once the experiment had ended, after recording the ethanol intakes at the second day (day 63) of alcohol relapse, the animals were anesthetized with chloral hydrate (280 mg/kg, i.p.) and euthanized to obtain hippocampal samples for determination of GSSG and GSH and astrocyte and microglia immunoreactivity.

It is important to note that drug administration in this study was always conducted in the daylight part of the circadian cycle. The relapse intake after ethanol reaccess [alcohol deprivation effect (ADE)] was started at 1:30 PM to 3:30 PM to dissociate ethanol intake from food consumption (lights on at 7 AM and off at 7 PM).

### Experiment 3. Effect of ASA or Lipoxin A4 Administration on Chronic Ethanol Intake: Role of the Formyl Peptide Receptor 2/Lipoxin A4 (FPR2/ALX)

Twenty female UChB rats, weighing 190–210 g, were single housed in cages at the age of 60 days and maintained on a 12-h light–dark cycle (lights off at 7:00 PM). Subsequently, rats received continuous concurrent free-choice access in the home cage to 10% (vol/vol) ethanol solution and water for 87 consecutive days. On day 88, rats were allowed to concurrent three-bottle choice access to 10% and 20% (vol/vol) ethanol solutions and water for 19 additional days. On day 102, rats were divided into five groups (five rats/group) that received for three consecutive days: (1) vehicle/ASA group: rats were given vehicle into the tail vein (i.v.) 5 min before ASA administration (15 mg/kg per day) given by oral gavage; (2) lipoxin (LXA)/vehicle group: rats were given vehicle (i.v.) 5 min before LXA administration (7.0 μg/kg per day) into the tail vein (Wu et al., 2013), an ASA-triggered endogenous lipid mediator with potent anti-inflammatory properties agonist of the FPR2/ALX receptor (Medeiros et al., 2013; Hughes et al., 2017); (3) WRW4/ASA group: rats were given WRW4, a FPR2/ALX receptor antagonist (0.3 mg/kg per day, i.v.; Sordi et al., 2013) 5 min before ASA administration (15 mg/kg per day, by oral gavage); (4) WRW4/vehicle group: rats were given WRW4 (0.3 mg/kg per day i.v.) 5 min before vehicle administration (water 5 ml/kg per day, by oral gavage); and (5) vehicle/vehicle group: rats were given vehicle into the tail vein (i.v.) 5 min before vehicle administration (water 5 ml/kg per day, by oral gavage). Ethanol intake was recorded daily.

### Experiment 4. Effect of the Administration of ASA on the Relapse-Like Ethanol Drinking: Role of the Formyl Peptide Receptor 2/Lipoxin A4 (FPR2/ALX)

Twenty female UChB rats, weighing 190–210 g, were single housed in cages at the age of 60 days and maintained on a 12-h light–dark cycle (lights off at 7:00 PM). Subsequently, rats received continuous concurrent free-choice access in the home cage to 10% (vol/vol) ethanol solution and water for 56 consecutive days. On day 57, rats were allowed three-bottle choice access to 10% and 20% (vol/vol) ethanol solutions and water for 17 additional days. On day 74, after induction of chronic ethanol intake, animals were deprived of ethanol for 14 days and thereafter allowed reaccess to ethanol solutions (10% and 20%), for 2 days. Water was always available throughout the experiment. During the last 3 days of ethanol deprivation, rats were divided into the following four groups (5 rats/group): (1) vehicle/ASA group: rats were given vehicle (isotonic saline; 1 ml/kg per day i.v.) 5 min before ASA administration (30 mg/kg per day, by oral gavage); (2) WRW4/ASA group: rats were given WRW4 (0.3 mg/kg per day i.v.), an antagonist of FPR2/ALX receptor (Sordi et al., 2013) 5 min before ASA administration (30 mg/kg per day, by oral gavage); (3) WRW4/vehicle group: rats were given WRW4 (0.3 mg/kg per day i.v.) 5 min before water administration (5 ml/kg per day, by oral gavage); (4) vehicle/vehicle group: rats were given vehicle (isotonic saline, 1 ml/kg per day, i.p.) 5 min before water administration (5 ml/kg per day by oral gavage).

### Statistical Analyses

Statistical analyses were performed using GraphPad Prism (San Diego, CA, USA). Data are expressed as means ± SEM. The normal distribution of data for all experiments was first tested using the Shapiro–Wilk test. For normally distributed data, one-way (Figures 1, 2, 4, 5, 6) or two-way (Figures 3, 7, 8) analysis of variance (ANOVA) was used followed by a Tukey or Fisher *post hoc* test. When only two groups were compared, statistical significance was determined by Student’s *t*-test. A level of *P* < 0.05 was considered for statistical significance.

To facilitate text reading, full statistical ANOVAs are presented in the legends to Figures.

## Results

Figure 1A shows that chronic ethanol intake of control animals (vehicle-treated; 16.0 ± 0.3 g/kg per day; mean ± SEM, *n* = 6) was inhibited by 45% by NAC administration (8.9 ± 0.3 g/kg per day; mean ± SEM; *n* = 6) and inhibited by 33% by ASA (administration (10.7 ± 0.5 g/kg per day; mean ± SEM, *n* = 6). Ethanol intake of animals receiving both NAC and ASA (NAC + ASA; 5.5 ± 0.1 g/kg per day; mean ± SEM, *n* = 6) was decreased by 66% vs. that shown by vehicle-treated animals (*P* < 0.001) and lower than that shown by rats receiving either NAC or ASA alone (*P* < 0.001). In Figure 1A, chronic ethanol intake (10 days–baseline) prior to vehicle administration was not different [not statistically significant (N.S.)] from that of rats receiving vehicle (white bar vs. blue bar). Figure 1B shows that NAC, ASA, and NAC + ASA administration markedly increased water intake compared to that shown by the control (ethanol vehicle) and baseline groups. Figure 1C shows that the administration of NAC, ASA, or the combination of NAC and ASA did not affect total fluid intake or body weight compared to vehicle administration.

Figure 2 shows the administration of NAC (40 mg/kg per day) markedly increased the expression in the hippocampus of a number of genes known to respond to Nrf2 activation of the ARE (Kansanen et al., 2013): (A) HO-1; (B) NAD(P)H:quinone oxidoreductase 1 (Nqo1); (C) GCLC; and (D) Prdx. Acetylsalicylic acid administration (15 mg/kg per day) did not raise the expression of HO-1, Nqo1, GCLC, or Prdx.

A separate experiment was conducted to determine the mechanism(s) by which NAC inhibits alcohol relapse intake (ADE), addressing the role played on ethanol intake by the xCT cystine-glutamate transporter and by the glutamate mGlu2/3 inhibitory receptor on alcohol relapse intake. In this experiment, rats were allowed to ingest alcohol for 48 days and subsequently alcohol-deprived for 14 days. After the deprivation period, alcohol reaccess (10% and 20% vol/vol) was allowed on days 62 and 63. Water was always available throughout the experiment. Figure 3 shows the complete loss of the NAC-induced inhibition of alcohol relapse intake afforded by the i.p. administration of both the inhibitor of the xCT transporter SZ (8 mg/kg per day i.p; Bernabucci et al., 2012) or the mGlu2/3 receptor inhibitor LY341495 (1 mg/kg per day i.p.; Fukumoto et al., 2014). These inhibitors were administered 15 min prior to NAC (100 mg/kg per day) administration, given by oral gavage, on the last 4 days of ethanol deprivation (days 58, 59, 60, and 61) 24 h prior to ethanol reaccess. On days 62 and 63, alcohol reaccess was allowed. Upon ethanol reaccess, the intake by the control group (e.g., vehicle/vehicle group) was on the first day of ethanol reaccess (13.3 ± 0.8 g/kg per day, *n* = 5; day 62) significantly higher (*P* < 0.05) than the intake prior to the 14-day deprivation period (10.5 ± 0.9 g/kg per day, *n* = 5). The administration of NAC inhibited ethanol intake by 70% to 75% (*P* < 0.01) both on days 62 and 63, an effect that was fully abolished (*P* < 0.001) by the administration of the xCT inhibitor SZ (8 mg/kg per day) or by the metabotropic Glu2/3 receptor antagonist LY341495 (1 mg g/kg per day).

As indicated above, an *in vitro* Nrf2 gene overexpression leads to increases in xCT gene expression (Shih et al., 2003; Habib et al., 2015). In the present study, NAC (100 mg/kg per day, administered on the last 4 days of the 14-day ethanol deprivation period) led to a small but significant increase (24%) in xCT mRNA level (1.1 ± 0.05 × CT mRNA/GADPH mRNA level; mean ± SEM, *n* = 6) compared to vehicle-treated rats (0.90 ± 0.04 × CT mRNA/GADPH mRNA level; mean ± SEM, *n* = 6; Student’s *t*-test: 2.437; *P* < 0.03).

It has been reported that upon alcohol relapse intake following deprivation (ADE), UChB rats display a binge-like drinking behavior within the first hour of alcohol reaccess (Israel et al., 2019; Quintanilla et al., 2019). It is noted that Figure 3 shows the daily (24-h) intake that follows the deprivation and ethanol reaccess. Figure 4 shows the relapse of ethanol drinking for the above animal set evaluated during the first 60 min of ethanol reaccess. In the first hour following ethanol reaccess, rats consumed 1.58 ± 0.09 g ethanol/kg, *n* = 6, intake that was inhibited by 75% to 80% (*P* < 0.01) by NAC (0.35 ± 0.19 g ethanol/kg, *n* = 6) administration. Both the xCT transporter inhibitor SZ and the mGlu2/3 receptor inhibitor LY341495 administered 15 min prior to the oral administration of NAC (100 mg/kg) fully blocked the inhibitory effect of NAC on ethanol intake (*P* < 0.001). Noteworthy, by itself, the mGlu2/3 receptor inhibitor LY341495 or the xCT transporter inhibitor SZ did not inhibit the binge-like relapse ethanol intake.

The mechanism of the strong antioxidant effect of NAC has not been fully elucidated. Conceivably, NAC can: (a) directly increase intracellular cysteine for the synthesis of intracellular glutathione; (b) generate glutathione following the Nrf2-mediated increase in γ-glutamyl-cysteine synthetase, a rate-limiting enzyme in the synthesis of glutathione (Lu, 2013); and/or (c) generate glutathione following an xCT-mediated transport of extracellular cystine into the cells. Figure 5 shows that the marked 2-fold to 3-fold increase in the hippocampal GSSG/GSH ratio induced by chronic alcohol intake (*P* < 0.01) was fully reversed by the administration of NAC (100 mg/kg per day per o.s. for 3 days; *P* < 0.01). Sulfasalazine administration fully reversed (*P* < 0.01) the effect of NAC on the GSSG/GSH ratio, supporting the view that the xCT-mediated influx of cystine into the cells is required to provide cysteine for the synthesis of glutathione. The mGlu2/3 receptor inhibitor LY341495 did moderately, but not significantly reduce the inhibitory effect of NAC on GSSG/GSH levels.

As indicated previously, a number of studies have shown that chronic alcohol treatment leads to neuroinflammation. Figure 6 shows that chronic ethanol intake even if followed by an alcohol deprivation period and relapse leads to significant increase in astrocyte activation evidenced by an increase in the length and thickness of astrocyte processes (GFAP immunoreactivity) and by an increase in microglial density (Iba-1 immunoreactivity). Unexpectedly, the restoration of the normal levels of astrocyte activation and microglial density afforded by NAC administration was fully blocked by both SZ and LY341495, suggesting that the glutamatergic tone plays a role in the generation and/or perpetuation of neuroinflammation.

As indicated earlier, ASA has been shown to induce remarkable anti-inflammatory effects *via* the generation of lipoxin A4 from arachidonic acid (also referred to ASA-triggered lipoxin or ATL-lipoxin). Figure 7 shows that the administration of both ASA and lipoxin A4 inhibited chronic alcohol intake, effects that were fully blocked (*P* < 0.001) by administration of WRW4, the antagonist of the lipoxin A4 receptor (FPR2).

Figure 8 shows that the administration of the antagonist of the lipoxin A4 receptor FPR2/ALX (WRW4) also blocked the inhibitory effect of ASA on relapse alcohol intake. In these studies, rats were offered free-choice access to 10% (vol/vol) ethanol solution and water for 56 consecutive days. On day 57, rats were allowed three-bottle choice access to 10% and 20% (vol/vol) ethanol solutions and water for 17 additional days. On day 74, animals were deprived of ethanol for 14 days and thereafter were allowed reaccess to ethanol solutions (10% and 20%) for 2 days. Water was always available throughout the experiment. During the last 3 days of ethanol deprivation, on days 85, 86, and 87, rats were divided in four groups: (1) vehicle/ASA group: received vehicle (isotonic saline; 1 ml/kg per day i.v. 5 min prior to ASA administration at 30 mg/kg per day, by oral gavage); (2) WRW4/ASA group: received WRW4 (0.3 mg/kg per day i.v. 5 min prior to ASA administration at 30 mg/kg per day, by oral gavage); (3) WRW4/vehicle group received WRW4 (0.3 mg/kg per day i.v. 5 min prior to vehicle (water) administration by oral gavage; and (4) vehicle/vehicle group received vehicle [isotonic saline i.v. 5 min before vehicle (water) administration by oral gavage]. On days 88 and 89 (24 h after discontinuation of all treatments), reaccess to ethanol (10% and 20% vol/vol solutions) was allowed. Water was always available. As can be seen, a 50% to 55% of the ASA-induced reduction of ethanol relapse was blocked (*P* < 0.05) by the administration of WRW4, an antagonist of FPR2/ALX receptor.

## Discussion

The present study shows that different mechanisms are involved in the inhibition of chronic alcohol intake/relapse drinking induced by NAC and ASA, which allows the combined administration of low doses of both drugs to achieve a larger inhibition of chronic alcohol intake/alcohol relapse, of possible relevance for the treatment of alcohol use disorders (AUDs).

As shown in the present studies, alcohol-induced neuroinflammation and oxidative stress remain for very long periods after chronic ethanol intake is discontinued, which along with the presentation of long-lasting conditioned cues, lead to relapse, characteristic of the chronicity of AUD (alcohol withdrawal removed in *Diagnostic and Statistical Manual of Mental Disorders, Fifth Edition*). Acetylsalicylic acid and NAC would be most valuably in a clinical setting to prevent relapse (patient being abstinent) rather than to reduce the alcohol intake of a patient actively consuming alcohol, also avoiding any untoward effects of the combination of ethanol and ASA.

Often, in therapeutic approaches using NAC, a loading dose is recommended, followed by lower maintenance doses (Fisher and Curry, 2019). The loading dose of 70 mg/kg administered in the present study is one-half of the Food and Drug Administration–recommended loading dose of NAC for the treatment of acetaminophen (paracetamol) liver injury. After the 2-day administration, blood levels of NAC (half-life 5.6 h: Sansone and Sansone, 2011) would be still below those achieved after a single clinical loading dose of 140 mg/kg. In translational terms, the administration of a loading dose by a health professional would allow determining whether drug hypersensitivity (present in 2%–3% of the general population) is seen. It may also deter AUD individuals from self-administration of over-the-counter sold NAC (available in many countries, including the United States, Canada, France, Italy, and Germany).

The maintenance dose of NAC of 40 mg/kg per day administered along with ASA (15 mg/kg per day) for 11 days reduced chronic ethanol intake by two-thirds. When NAC was administered at dose of 100 mg/kg per day (*per os*) for only 4 days (to match the schedule of administration of LY341495 or SZ) during the alcohol deprivation period, a marked inhibition (75% to >80%) was observed on: (i) alcohol relapse; (ii) oxidative stress; and (iii) neuroinflammation. The effect of NAC on alcohol relapse and neuroinflammation was fully reversed by the xCT inhibitor SZ (8 mg/kg per day). These results support the suggestion (Lebourgeois et al., 2019) that NAC might be effective in reducing ethanol intake by activation of the xCT -mediated glutamate transporter. The mGlu2/3 receptor inhibitor LY341495 had only a marginal effect on reversing the GSSG/GSH ratio, indicating that lowering of oxidative stress induced by NAC is not primarily associated with the glutamatergic tone.

A note on the specificity of SZ is in order. At doses of 25, 50, and 100 mg/kg per day, SZ was reported to induce analgesic effects in an animal model of inflammatory pain, while in the same study a dose of 8 mg/kg per day showed no effects (Bernabucci et al., 2012). Such a dose (8 mg/kg per day) was chosen in this study. It was recently reported that SZ administered at 30 mg/kg per day was minimally effective on the treatment of inflammatory bowel disease in rats, whereas a marked effect required the coadministration of anti-inflammatory mesenchymal stem cells (Yousefi-Ahmadipour et al., 2019). In a recently developed model of inflammatory bowel disease in rat, a dose of SZ of 300 mg/kg per day was used to show therapeutic effects (Ghattamaneni et al., 2019). In an established model of arthritis in rats, SZ was administered chronically at a dose of 80 mg/kg per day (Fener et al., 1990). In the present study at the dose of 8 mg/kg per day, an anti-inflammatory effect *per se* was not observed by SZ.

The results shown by the present study strongly suggest that NAC administration inhibited ethanol-induced oxidative stress (GSSG/GSH ratio) activating the Nrf2-ARE system, which is in line with a report that an analog of NAC, NACA, attenuated the oxidative stress induced by a traumatic brain injury in rats *via* the same antioxidant system (Zhou et al., 2018). The Nrf2 system activates the transcription of a number of antioxidant enzymes, including HO, NAD(P)H, quinone oxidoreductase 1, Prdx, and γ-glutamyl-cysteine synthetase, the latter a rate-limiting enzyme in the synthesis of glutathione. An additional rate-limiting factor in the synthesis of glutathione is the availability of intracellular cysteine (Lu, 2013). The Nrf2 system additionally up-regulates the activity of the promoter of the xCT cystine-glutamate transporter gene (Shih et al., 2003; Habib et al., 2015), a transporter that allows the influx of cystine into cells by exchanging it with glutamate (Baker et al., 2002). In the present study, the expression of the xCT was marginally but significantly increased by NAC administration.

Whether an activation of xCT transporter function due to an increased substrate availability and/or an increased xCT expression plays a role in the NAC-induced reduction of hippocampal GSSG/GSH ratio, the administration of the xCT inhibitor SZ was expected to reduce or block such an effect of NAC. As indicated, this was indeed observed; SZ fully blocked the effect of NAC in lowering the GSSG/GSH ratio. This finding, added to the observation that SZ also fully blocked the NAC-induced inhibition of relapse-like ethanol intake, suggests that NAC acts *via* the xCT transporter extruding glutamate into the extracellular space (Baker et al., 2002) reaching levels known to stimulate presynaptic mGlu2/3 autoreceptors, negatively modulating synaptic glutamate release (Schoepp et al., 1999). This effect of NAC is of additional value for reducing ethanol intake because mGlu2/3 receptor has been reported to be depressed in animals chronically exposed to alcohol (Meinhardt et al., 2013; Ding et al., 2016). An increased mGlu2/3 receptor action by NAC is further supported by the finding in the present study that pretreatment of animals with the mGluR2/3 receptor antagonist, LY341495, fully blocked the NAC-induced inhibition of alcohol relapse.

Operant ethanol self-administration studies by Gass et al. (2011) showed that the presentation of cues previously associated with alcohol self-administration led to marked increases in glutamate release in nucleus accumbens. An increased synaptic release of glutamate appears to play an important role in addictive drug craving and relapse for most drugs of abuse (Scofield et al., 2016). In the present ADE study, alcohol reaccess provides conditioned cues such as ethanol odor, reported in heavy alcohol users to activate nucleus accumbens (Kareken et al., 2004) and taste (Remedios et al., 2014). An mGlu2/3 receptor-mediated inhibition of the glutamate tone by NAC is expected to improve the effect when combined with an increased synaptic GLT-1 transport, as previously reported for ASA (Israel et al., 2019). N-acetylcysteine is expected to have a synergic effect on the inhibition of alcohol intake induced by β-lactam antibiotics such as ceftriaxone and ampicillin, which increase GLT-1 activity (Rao et al., 2015; Sari et al., 2016). Such a synergic effect with NAC may be of value in the use of central nervous system–directed β-lactam antibiotics in clinical studies.

The doses of NAC and ASA used to reduce alcohol intake in the present study are within the range used for clinical studies. N-acetylcysteine at doses among 2,400–3,600 mg/day have been used for the treatment of cocaine, nicotine, alcohol, and cannabis relapse in humans (Duailibi et al., 2017; Squeglia et al., 2018). The 15 or 30 mg/kg per day dose of ASA used does not induce gastric irritation in the rat (Wallace et al., 2004). The average daily dose of ASA used chronically in the treatment of rheumatoid arthritis is 2,600 mg/day (Fries et al., 1993). Thus, in humans, doses of ASA could be markedly increased vs. those used in this study. The combination of NAC (1,000 mg/day) plus ASA (1,000 mg/day) has been administered with positive results in the treatment of bipolar depression (Bauer et al., 2019).

A possible depressant effect in the animals in the present study, in which the rats were single housed without environmental enrichment, is unlikely as a cause of neuroinflammation, because as shown in Figure 6, control animals housed individually and receiving water as the only fluid do not show astrocyte or microglial abnormalities. Nevertheless, the existence of nonhistological abnormalities, or stress often present in AUD, cannot be discarded and may have a role in the inhibition of neuroinflammation exerted by the combination of NAC and ASA (see Bauer et al., 2019).

A methodological aspect of interest relates to the minimal length of chronic alcohol intake required, by a model of spontaneous alcohol intake, to observe a marked effect of NAC. It is important to use a model where NAC (at the doses used) has a clear inhibitory effect on ethanol intake, such that a possible association with neuroinflammation or oxidative stress can be investigated. We have reported that NAC does not inhibit alcohol intake if rats are allowed ethanol intake for a short period (20 days; Quintanilla et al., 2016b), time at which an ethanol intake plateau had not been reached. However, in the same study, an inhibitory effect of NAC on ethanol intake was clearly observed following 60 days of chronic alcohol intake, time at which intake was constant. In the present study, chronic ethanol intake for 47 days (36 days of 10% ethanol access plus 11 days of 10% and 20% ethanol access), followed by a 14-day deprivation period, showed a marked relapse intake, which was inhibited by NAC (day 62). An inhibitory effect of NAC at such time is in line with a self-perpetuating neuroinflammation even after ethanol deprivation. The present work with UChB rats shows the shortest time (voluntary intake plus deprivation) that has even been reported for rats showing neuroinflammation. In previous studies, the authors have shown alcohol-induced neuroinflammation after longer times of ethanol access (>91 days). Studies in mice (Alfonso-Loeches et al., 2010) that had ingested 10% alcohol for 150 days showed significant neuroinflammation both in cerebral cortex and hippocampus. Whether shorter times will also show an ethanol-induced neuroinflammation is not known.

As indicated earlier, ASA acutely acetylates Cox-1 and chronically also Cox-2, leading to the generation of the powerful anti-inflammatory 15- R epi-Lipoxin A4 (or ASA triggered lipoxin ATL), a metabolite of arachidonic acid (Romano et al., 2015; Serhan and Levy, 2018). The present study shows that the administration of lipoxin A4 partially inhibited chronic alcohol intake. Furthermore, the inhibitory effect of ASA on alcohol relapse intake was significantly reduced by the administration of WRW4, an antagonist of the lipoxin receptor FPR2/ALX, suggesting that the generation of lipoxin is the main mechanism behind the reduction of chronic alcohol intake induced by ASA. To our knowledge, this is the first report of the inhibitory effect of lipoxin A4, a macrophage recruitment inhibitor (Serhan and Levy, 2018) on alcohol intake.

Overall, the present study shows that NAC and ASA inhibit alcohol intake by different mechanisms, resulting in a combined effect that could allow the chronic use of both drugs at clinically relevant doses.

## Data Availability Statement

All datasets presented in this study are included in the article.

## Ethics Statement

The animal study was reviewed and approved by Medical Faculty of the University of Chile (Protocol CBA# 0994 FMUCH) and by the Chilean Council for Science and Technology Research (CONICYT).

## Author Contributions

MQ and FE: conception and design, collection of data, data analysis, manuscript writing, and final approval of manuscript. PM: design, collection of data, data analysis, and final approval of manuscript. ME: conception and design, collection of data, data analysis, financial support, manuscript writing, and final approval of manuscript. BO and DS: collection of data, data analysis, and final approval of manuscript. MH-M: conception and final approval of manuscript. YI: conception and design, financial support, manuscript draft and final approval of manuscript.

## Conflict of Interest

The authors declare that the research was conducted in the absence of any commercial or financial relationships that could be construed as a potential conflict of interest.
